# Complex interplay among fat, lean tissue, bone mineral density and bone turnover markers in older men

**DOI:** 10.18632/aging.103903

**Published:** 2020-10-09

**Authors:** Aleksandra Rył, Iwona Rotter, Aleksandra Szylińska, Alina Jurewicz, Andrzej Bohatyrewicz, Tomasz Miazgowski

**Affiliations:** 1 Department of Medical Rehabilitation and Clinical Physiotherapy, Pomeranian Medical University in Szczecin, Szczecin, Poland; 2 Department of Orthopaedics Traumatology and Musculoskeletal Oncology, Pomeranian Medical University in Szczecin, Szczecin, Poland; 3 Department of Propedeutics of Internal Medicine and Arterial Hypertension, Pomeranian Medical University in Szczecin, Szczecin, Poland

**Keywords:** bone mineral density, body composition, bone turnover markers, older men

## Abstract

It has been suggested that visceral fat (VF) might be a negative determinant of bone health. The purpose of this cross-sectional study was to assess an interplay among fat, visceral fat (VF), muscle mass, bone mineral density (BMD), and markers of bone turnover in men aged 60-75 years. BMD, lean mass, total fat, VF and appendicular skeletal muscle mass (ASM) were assessed using dual-energy X-ray absorptiometry. Using ELISA assays, we measured serum levels of markers of bone turnover (osteocalcin, parathyroid hormone, human procollagen I N-terminal peptide, and degradation products of C-terminal telopeptide of type I collagen). Mean values of bone markers were within normal range. VF was found not to be associated with BMD and bone turnover markers. ASM was inversely correlated with age, and positively with BMD and lean mass. In linear regression, ASM, VF, total fat, lean mass and body mass index were significant single predictors of BMD. However, after adjustment for age, all these associations were no longer significant. In conclusion, in contrast to some studies on postmenopausal women, in older non-diabetic men with normal lean mass and body fat VF was not associated with BMD and markers of bone formation and resorption.

## INTRODUCTION

It has been well established that lifetime bone mass in humans is strongly determined by age, sex, hormonal milieu, genetic influences, and lifestyle. Bone mass, routinely assessed using dual-energy X-ray absorptiometry (DXA) as bone mineral density (BMD), is correlated with body mass index (BMI) [1, 2] and other anthropometric measures [3] as well as body composition, although the impact of fat and lean tissue on BMD may vary [4]. Lean tissue, which is mainly composed of muscles, exerts a strong positive effect on bone mass [5, 6], while the impact of fat is weaker and likely indirect [7, 8]. Much less is known about the precise associations of BMD with regional fat depots. Of them, visceral fat (VF)–an important contributor to various metabolic disorders–could theoretically influence bone metabolism as it is involved in the development of insulin resistance and related cardiometabolic disorders. Indeed, in postmenopausal women VF was found to be a negative determinant of BMD and markers of bone turnover [9], as well as poor bone microarchitecture and weakened bone strength [10, 11] and these effects were stronger than those of other fat regions. However, it remains uncertain whether these associations apply to adult males.

The purpose of this cross-sectional study was to assess an interplay among total fat, VF, muscle mass, BMD, and markers of bone formation and resorption in older men.

## RESULTS

Descriptive statistics of the study participants are shown in Table 1. The mean age of the sample was 66.18 ± 5 years and BMIs ranged from 20.4 to 39.4 kg/m^2^. The mean values of markers of bone formation (osteocalcin; human procollagen I N-terminal peptide [PINP]) and resorption (parathyroid hormone [PTH]; degradation products of C-terminal telopeptide of type I collagen [CTX-I]) were within normal reference ranges. Likewise, lean mass (sex-specific reference values: 58.9 ± 7 kg and 54.3 ± 6 kg for men aged 60-69 and 70-70 years, respectively [12]) and body fat percentage (sex- and age-specific reference value: 31 ± 7% [13]) were normal. VF was a relatively small component of the body and accounted for only 2.75% of weight and 7.9% of total fat. The majority of the patients had normal BMD and 11 patients had osteopenia (hip t-score between -1 to -2.5 standard deviation [SD]). None of the study participants had osteoporosis defined as a total hip t-score below -2.5 SD.

**Table 1:** Patient characteristics.

	**mean ± SD**	**Range**	**Median**
Anthropometric measures			
Age (years)	66.18 ± 5.04	60.0-75.0	66.0
Height (cm)	174.9 ± 6.31	158.0-190.0	175.0
Weight (kg)	90.88 ± 14.95	51.0-130.0	90.0
BMI (kg/m^2^)	29.52 ± 3.98	(20.43-39.45)	29.4
Bone mineral density			
Spine (g/cm^2^)	1.43 ± 0.23	1.01-2.10	1.40
Total hip (g/cm^2^)	1.25 ± 0.15	0.99-1.56	1.26
Total hip (t-score)	0.52 ± 1.46	-2.10-3.60	0.60
Body composition			
Lean mass (kg)	55.04 ± 6.77	39.94-74.50	56.33
Total fat (kg)	31.57 ± 6.99	13.89-47.28	32.2
Bone mineral content (kg)	3.05 ± 0.40	2.09-4.01	3.03
Visceral fat (kg)	2.50 ± 0.88	0.40-4.53	2.57
Visceral fat (cm^3^)	2657.5 ± 922	429-4804	2614.0
ASM (kg)	24.56 ± 38.77	16.65-40.96	24.55
Markers of bone turnover			
OC (ng/ml)	6.44 ± 4.23	0.20-29.94	5.69
PTH (pg/ml)	36.36 ± 22.46	2.70-161.3	31.95
CTX-I (ng/ml)	0.45 ± 0.22	0.06-1.80	0.41
PINP (ng/ml)	961.23 ± 940.38	5.82-3689.3	811.0

ASM – appendicular skeletal muscle mass; OC – osteocalcin; PTH – parathyroid hormone; CTX-I – C-terminal telopeptide of type I collagen; PINP – human procollagen I N-terminal peptide

Next, we compared BMD and bone turnover markers in groups with ASM, VF and total fat below and above the median value, and BMI below and above a cut-off of 30 kg/m^2^ (Table 2). Patients with ASM above the median had bone mineral content (BMC) higher by 17% and hip BMD higher by 13%, but similar values of bone turnover markers (both bone formation and resorption) in comparison to those with ASM were below the median. No significant differences in bone turnover markers and BMD were found between the groups divided according to the medians of VF, total fat and BMI.

**Table 2:** Intergroup analysis with *ASM,* BMI, total and visceral fat, and bone turnover markers above and below the median value.

	**ASM**			**Total fat**			**Visceral fat**			**BMI**		
	**below median n = 58**	**above median n = 84**	**p**	**below median n = 61**	**above median n = 81**	**p**	**below median n = 56**	**above median n = 86**	**p**	**< 30 kg/m^2^ n = 74**	**> 30 kg/m^2^ n = 68**	**p**
Age (years)	66.59 ± 4.46 (66.0-75.0)	64.96 ± 4.75 (65.0-75.0)	0.199	66.22 ± 4.94 (65.0-75.0)	65.33 ± 4.43 (60.0-75.0)	0.561	66.30 ± 5.08 (60.0-75.0)	65.26 ± 4.17 (60.0-75.0)	0.416	66.65 ± 4.99 (60.0-75.0)	65.58 ± 5.09 (60.0-75.0)	0.230
**Markers of bone turnover**												
PINP (ng/ml)	1380.4 ± 1086 (891-3689)	1361.1 ± 1104 (908-3519)	0.689	1349.4 ± 1123 (87.64-3689)	1391.6 ± 1067 (85.82-3599)	0.656	1359.2 ± 1121 (87.64-3689)	1382.2 ± 1069 (85.82-3599)	0.939	1086.1 ± 980 (35.82-3689)	797.01 ± 865 (5.82-3599)	0.089
CTX-I (ng/ml)	0.52 ± 0.23 (0.43-1.07)	0.43 ± 0.16 (0.41-0.91)	0.949	0.44 ± 0.19 (0.13-0.78)	0.50 ± 0.21 (0.24-1.07)	0.563	0.45 ± 0.18 (0.13-0.78)	0.49 ± 0.22 (0.23-1.07)	0.474	0.45 ± 0.25 (0.06-1.80)	0.43 ± 0.17 (0.15-0.91)	0.634
PTH (pg/ml)	37.83 ± 23.97 (32.6-118.08)	36.66 ± 19.17 (33.24-90.82)	0.104	34.24 ± 13.13 (15.98-74.37)	39.88 ± 26.77 (4.04-118.08)	0.792	34.71 ± 12.7 (15.98-74.37)	39.47 ± 27.02 (4.05-118.08)	0.435	37.07 ± 23.21 (2.70-161.35)	35.42 ± 21.6 (4.05-110.27)	0.686
OC (ng/ml)	6.99 ± 3.91 (6.27-16.05)	6.12 ± 3.96 (5.55-15.77)	0.847	6.94 ± 3.92 (1.08-16.05)	6.19 ± 3.96 (0.20-15.77)	0.355	7.05 ± 3.87 (1.08-16.05)	6.09 ± 3.98 (0.20-15.77)	0.378	6.96 ± 4.77 (0.40-29.94)	5.75 ± 3.30 (0.20-15.77)	0.110
**Bone mineral density**												
Total hip (g/cm^2^)	1.18 ± 0.13 (1.19-1.41)	1.33 ± 0.14 (1.31-1.56)	0.002	1.24 ± 0.14 (1.01-1.55)	1.27 ± 0.15 (0.99-1.56)	0.483	1.23 ± 0.15 (0.99-1.55)	1.28 ± 0.15 (1.01-1.56)	0.256	1.22 ± 0.16 (0.99-1.55)	1.30 ± 0.13 (1.04-1.56)	0.061
Total hip (t-score)	0.33 ± 1.45 (-2.10-3.60)	0.71 ± 1.36 (-1.1-3.40)	0.301	0.43 ± 1.39 (-1.19-3.40)	0.52 ± 1.45 (-2.10-3.60)	0.682	0.35 ± 1.47 (-2.10-3.40)	0.70 ± 1.44 (-1.90-3.60)	0.493	0.09 ± 1.24 (-2.10-3.40)	0.32 ± 1.52 (-2.10-3.60)	0.314
BMC (kg)	2.81 ± 3.07 (2.90-3.19)	3.29 ± 3.25 (3.32-4.01)	0.003	3.00 ± 4.37 (2.08-4.01)	3.05 ± 3.95 (2.08-4.01)	0.598	2.99 ± 4.44 (2.08-4.01)	3.10 ± 3.40 (2.35-3.84)	0.312	2.996 ± 4.52 (2.08-4.01)	3.12 ± 2.96 (2.69-3.76)	0.239

Data are presented as means ± SD and ranges. ASM – appendicular skeletal muscle mass; OC – osteocalcin; PTH – parathyroid hormone; CTX-I – C-terminal telopeptide of type I collagen; PINP – human procollagen I N-terminal peptide; BMC – bone mineral content

Similarly, in correlation analysis (Table 3), VF, total fat and BMI were not associated with any bone turnover markers. As expected, ASM was inversely correlated with age and positively with lean mass, BMD (both spine and hip), and BMC. Lean mass significantly correlated with spine BMD (R = 0.537; p < 0.001) and hip BMD (R = 0.648; p < 0.001). Interestingly, total fat was positively associated only with spine BMD, while VF, similarly to BMI, was associated neither with bone mass nor bone turnover.

**Table 3:** Correlations between ASM, VAT, TF, BMI, markers of bone turnover and BMD.

	**ASM**		**Total fat**		**Visceral fat**		**BMI**	
	**R**	**p**	**R**	**p**	**R**	**p**	**R**	**p**
Age (years)	-0.468	0.005	-0.148	0.286	-0.021	0.903	-0.200	0.256
PINP (ng/ml)	0.101	0.568	0.088	0.531	0.100	0.572	0.056	0.749
CTX-I (ng/ml)	-0.179	0.311	-0.052	0.713	-0.166	0.358	-0.015	0.927
PTH (ng/ml)	0.039	0.825	0.040	0.781	0.104	0.557	0.226	0.199
OC (ng/ml)	-0.093	0.600	-0.168	0.229	-0.174	0.318	-0.041	0.817
Spine (g/cm^2^)	0.423	0.013	0.294	0.031	0.228	0.194	0.329	0.057
Hip (g/cm^2^)	0.532	0.001	0.177	0.200	0.113	0.521	0.345	0.045
Hip (t-score)	0.579	0.003	0.244	0.079	0.166	0.347	0.450	0.008
Lean mass (kg)	0.426	0.001	-0.044	0.752	-0.126	0.366	0.139	0.318
BMC (kg)	0.638	0.001	0.256	0.061	0.233	0.185	0.378	0.027

ASM – appendicular skeletal muscle mass; OC – osteocalcin; PTH – parathyroid hormone; CTX-I – C-terminal telopeptide of type I collagen; PINP – human procollagen I N-terminal peptide; BMC – bone mineral content.

As shown in Table 4, in linear regression, ASM, VF, total fat, lean mass, and BMI were single predictors of spine BMD and hip BMD (except of VF). However, after adjustment for age all these associations were no longer significant.

**Table 4:** Associations between BMD and ASM, visceral and total fat, lean mass, and BMI.

**Bone mineral density**		**beta**	**95% CI (lower)**	**95% CI (upper)**	**p**
Spine	ASM	0.460	0.214	0.708	0.001
	Visceral fat	0.315	0.052	0.580	0.020
	Total fat	0.388	0.132	0.645	0.004
	Lean mass	0.537	0.302	0.772	0.001
	BMI	0.437	0.185	0.691	0.001
Hip	ASM	0.601	0.379	0.823	0.001
	Visceral fat	0.199	-0.073	0.472	0.148
	Total fat	0.336	0.075	0.599	0.013
	Lean mass	0.648	0.436	0.860	0.001
	BMI	0.436	0.183	0.689	0.001

## DISCUSSION

In this study, we report for the first time the effects of VF on skeleton in the population of older non-diabetic and non-osteoporotic men. Our results suggest that in this population bone mass and turnover are normal regardless of BMI, total fat, and VF mass. Bone mass is determined by a wide range of genetic, hormonal and environmental factors. Vast majority of earlier studies demonstrated that obesity, particularly in postmenopausal women, could be potentially protective for the skeleton due to the effects of skeletal loading by increased fat mass, increased peripheral conversion of estrogen to androgens in the adipose tissue and higher level of circulating sex hormone binding globulin (SHBG) [14], leptin [15] and insulin [16], which altogether may work to stimulate osteoblast differentiation. Despite these observations, some studies have questioned the protective effects of increased body mass on skeletal health demonstrating that obesity decreases the risk of fractures [17] and that the contribution of obesity to fracture risk can be different in males and females [18].

Similarly, there have been conflicting reports on the associations of VF with bone health. It has been suggested that that excess VF might be detrimental to bone architecture and strength [9, 10] even when BMI is normal [11]. On the other hand, Liu et al. [19] found in postmenopausal females from the Framingham Offspring cohort that higher amount of VF was associated with greater BMD and better microstructure of the peripheral skeleton. However, these associations were not significant after adjustment for BMI, suggesting that the effects of VF on bone were driven by mechanical loading of weight rather than metabolic consequences of excess VF. In the other study on postmenopausal women, VF was an independent positive predictor of BMD; however, the direction of this association changed to negative after controlling for BMI, suggesting that VF rather than BMI could be a determinant of bone health [9]. In our study, VF and BMI were single predictors of BMD but the associations were no longer significant after adjustment for age. These discrepancies between the genders might be related to changes in hormonal milieu with aging: in contrast to menopause, a decline in testicular function can be initiated in any time point during middle adulthood and a decrease in testosterone level in aging men is usually gradual and less overt than a deficit of estrogens after menopause. In premenopausal women, higher VF was associated with lower bone formation and lower levels of insulin-like growth factor 1 [10, 20], while in men in their 30s with lower level of growth hormone - potent determinant of trabecular thickness [21], suggesting the different hormonal effects of VF on bone in younger men and women. Aside from potential hormonal differences, VF may affect the skeleton via metabolic effects. Excess VF is associated with higher insulin levels and the development of insulin resistance. Insulin in physiological concentrations exerts anabolic effects on bone tissue by increasing osteoblast proliferation rate and collagen synthesis, and decreasing osteoclast activity [22]. Recent study have shown that in the presence hyperinsulinemia and insulin resistance, BMD is greater and fracture rate the same as in individuals with preserved insulin sensitivity [23]. In this context, the effects of changes in sex hormones, growth hormone and other hormones, as well as weight and BMI on BMD might be escalated by VF-induced hyperinsulinemia. We found that ASM stronger than BMI, correlated with BMD and BMC. This finding supports the importance of preserving normal muscle mass through the lifespan: as the age-related decrease in muscle mass begins earlier than decrease in bone mass [24], normal ASM/muscle mass is essential in preventing both sarcopenia and osteoporosis.

We did not find any associations of VF with markers of bone turnover. The levels of markers of formation and resorption were within normal range and the levels did not differ when analyzed in groups divided by the median of ASM, total fat, VF and BMI. Moreover, none of markers was correlated with parameters of body composition. In contrast to our results, Sharma et al. [9] found in non-osteoporotic postmenopausal women that VF was a negative determinant of CTX-I and osteocalcin levels suggesting a different patterns in older males and females of bone markers response to excess VF.

Our study has some limitations. Firstly, we did not assessed smoking, alcohol consumption, dietary calcium intake and the level of physical activity, which are known to affect bone mass and quality. Secondly, we did not measure vitamin D levels. Finally, the study sample comprised non-diabetic men within a relatively narrow age range of 15 years. Therefore, our conclusions may not apply to other populations.

In conclusion, our study demonstrated that in contrast to some studies on postmenopausal women, in older men with normal lean mass and body fat percentage, VF, despite the well-recognized negative effects on metabolic health, was not associated with spine BMD and hip BMD, as well as markers of bone formation and resorption.

## MATERIALS AND METHODS

### Study participants

The study sample comprised 142 men aged 60-75 years, recruited from the patients who were referred for consultation due to chronic arthritis to our secondary care orthopedic surgery unit. The exclusion criteria included diabetes, malignant neoplasms, active alcohol disease, liver or renal insufficiency, heart failure class III or IV NYHA, the use of agents that may affect bone metabolism such as mineral supplements, neuroleptics, steroid drugs, and antidepressants. All subjects gave their informed consent for inclusion before they participated in the study. The study was conducted in accordance with the Declaration of Helsinki. The protocol of the study was approved by the Bioethical Commission of the Pomeranian Medical University in Szczecin (approval number KB-0012/155/16).

### Measurements of BMD and body composition

BMD and body composition were measured using DXA (Lunar Prodigy Advance; GE Healthcare; Madison, WI, USA). We measured BMD of the lumbar spine (L1-L4) and total hip. Using the automatic whole-body scan, we assessed lean mass, total fat mass and bone mineral content, and we calculated appendicular skeletal muscle mass as the sum of nonfat and nonbone tissue in both arms and legs [25, 26]. VF was assessed using the device-dedicated CoreScan^®^ application, which computes VF mass and volume from the difference of total and subcutaneous fat in the android region of interest.

### Markers of bone turnover

Using ELISA commercial assays (DRG MedTek; Warsaw, Poland), we measured serum levels of osteocalcin (normal range: 5-25 ng/ml), parathyroid hormone (normal range: 10-60 pg/ml), human procollagen I N-terminal peptide (normal range: 85.55-2028.75 ng/ml), and degradation products of C-terminal telopeptide of type I collagen (normal range: 0.115-0.748 ng/ml). All blood samples were collected in the morning after overnight fast. Serum was stored in Eppendorf tubes in a freezer at the temperature of -20°C.

### Statistical analysis

Statistical analysis was performed using SPSS Statistics, v. 13.1. (StatSoft, Inc. Tulsa, OK, USA). Data are presented as means, medians, standard deviations, and ranges. Normality of the distribution was assessed using the Shapiro-Wilk test. Variables with a normal distribution were compared using two-sided Student’s *t* test, otherwise non-parametric test were used. Correlations between pairs of quantitative variables were assessed using Pearson’s linear correlations or Spearman’s rho correlations for normally and non-normally distributed variables, respectively. Determinants of spine and hip BMD were tested using a simple linear regression models. Due to multicollinearity among DXA-derived body composition parameters, predictors of spine and hip BMD were calculated using ridge regression. The Lagrange multiplier (λ value), which controls the strength of the penalty term and minimizes the mean square error, was included in the final model. P-value less than 0.05 was considered statistically significant.
